# In Memoriam, Nathaniel M. Reichek, MD, 1941–2021

**DOI:** 10.1186/s12968-021-00804-6

**Published:** 2021-10-14

**Authors:** Christopher M. Kramer, Victor Ferrari

**Affiliations:** 1 University of Virginia Health System, Charlottesville, VA USA; 2 University of Pennsylvania, Philadelphia, PA USA

 Dr. Nathaniel Reichek, a founding father of the field of cardiovascular magnetic resonance (CMR), 3rd president of the Society for Cardiovascular Magnetic Resonance (SCMR), and 2017 recipient of the SCMR Gold Medal passed away in March 2021.

Nat received his undergraduate (summa cum laude, class salutarian) and MD (AOA) degrees from Columbia University and then trained in Medicine at Albert Einstein College of Medicine in New York. He completed his cardiology fellowship at Georgetown University, Washington, DC under the tutelage of Joseph Perloff MD and then followed his mentor to the University of Pennsylvania as junior faculty. He was at the University of Pennsylvania for 20 years, rising to the rank of Full Professor and director of the Noninvasive Laboratories. In 1992 he moved to Allegheny General Hospital as Chief of the Cardiology Division where he remained until 2002 at which time he took on the position as Director of the Research and Education Department at St. Francis Hospital, Rosyln, New York, USA and Professor of Medicine and Biomedical Engineering at SUNY Stony Brook. He stepped back to part-time status in 2016 to continue his research in CMR.

Nat was of service to SCMR since its inception. He was present when the Society was first proposed in a hotel conference room in Dallas, Texas during the 1994 American Heart Association (AHA) Annual Scientific Session. He was the 3rd President of SCMR and led the Society during a time when it was still finding its way, bridging a potential divide between Europe and the U.S. at a critical time in the Society’s development. He was a strong leader and did well at bringing disparate minds together. He was able to problem solve at a moment’s notice especially during the annual meeting, finding multiple last-minute replacement speakers and averting a potential crisis for the nascent Society. He had multiple other roles in the Society as well, including Chair of the Clinical Trials Committee, the Nominating Committee, the U.S. Reimbursement Subcommittee, the Publications Committee, and member of the US Chapter Executive Committee and the Mentorship Program. He worked hard on securing CMR reimbursement and other financial issues on behalf of the U.S. Chapter. He was a fixture within multiple international cardiology societies including the AHA, American College of Cardiology (ACC), and Intersocietal Commission for the Accreditation of Magnetic Resonance Laboratories (ICAMRL - now known as IAC-MRI), always strongly advocating for the interests of SCMR. For many years, he and the SCMR were practically synonymous. Nat was also a major contributor to the *Journal of the Society for Cardiovascular Magnetic Resonance (JCMR),* serving as an inaugural member of the editorial board, as a senior advisor for the past 5 years, and as our most frequent Guest Editor for conflict papers involving manuscripts submitted by the editorial team.

Nat contributed greatly to research in CMR for 3 decades. He was one of the first cardiac imagers, along with Drs. Gerald Pohost and Charley Higgins, to delve into the field in its very earliest days. One of his earliest contributions was the 1989 paper by Aurigemma et al. in *Circulation* “Noninvasive determination of coronary artery bypass graft patency by cine MRI” which used cine to document graft patency. His long-term collaboration with Dr. Leon Axel at the University of Pennsylvania is legendary as they published a series of manuscripts regarding the use of myocardial tagging to characterize intramural function, first in normal subjects and then in myocardial infarction, hypertrophic cardiomyopathy, and other disease states. He was involved over the years with many of the novel applications of CMR including tagging and late gadolinium enhancement. He was one of the principal investigators of the Women’s Ischemia Syndrome Evaluation (WISE) study that has made many contributions to our understanding of chest pain and coronary artery disease in women. He authored over 180 full peer-reviewed publications, many in the highest impact cardiovascular journals as well as more than 70 reviews and editorials. He was a leader in the cardiovascular research community. He chaired Cardiovascular A study section for the NHLBI/NIH several years after completing his term as a member. He also chaired AHA study section reviews. He was on the editorial boards of multiple cardiology journals including *Circulation* and *JACC* as well as *JCMR* and other subspecialty imaging journals.

Nat was a legendary diagnostician, and passed on the “Perloff Pearls” of physical examination to several generations of physicians. He volunteered to teach medical students and residents these skills throughout his career, and delighted in examining patients with unique findings that the fellows would try to “stump” him with. He was a master of the Socratic method at the bedside, and his skill in teaching deductive reasoning and medical decision-making was unmatched.

Arguably, Nat’s greatest contribution to the field of SCMR was his mentorship. He mentored 2 past presidents of the SCMR (Drs. Christopher Kramer and Victor Ferrari). His list of previous imaging trainees and junior faculty reads like a Who’s Who of cardiac imaging and includes Drs. Richard Devereux at New York Hospital; Pamela Douglas, past-President of the ACC; Joao Lima, SCMR Gold Medal Awardee and Director of Cardiovascular Imaging at Johns Hopkins; the late Martin St. John Sutton, former head of the Noninvasive Laboratories at the University of Pennsylvania; Gerard Aurigemma, Director of the Echocardiography Laboratory at the University of Massachusetts; and Jane Cao, Director of Cardiac Imaging at Catholic Health System of Long Island. He was a terrific teacher of research methodology and a highly skilled scientific writer and editor. He was an exemplary research mentor.

Nat Reichek was truly a quadruple threat in the field of CMR—a leader, researcher, clinician, and mentor. He will be sorely missed. In recognition of his many SCMR contributions and to sustain his legacy, the SCMR has named the SCMR Research and Educational Fund in his honor. For those who wish, contributions to the SCMR Nathaniel Reichek Research and Education Fund can be made at: https://scmr.org/donations/fund.asp?id=11805.

